# Characterization of the microbiota and volatile components of kocho, a traditional fermented food of Ethiopia

**DOI:** 10.1016/j.heliyon.2019.e01842

**Published:** 2019-06-08

**Authors:** Helen Weldemichael, Dominic Stoll, Christoph Weinert, Tesfemariam Berhe, Shimelis Admassu, Melaku Alemu, Melanie Huch

**Affiliations:** a School of Chemical and Bioengineering, Addis Ababa Institute of Technology, P.O. Box 385, Addis Ababa, Ethiopia; b Max Rubner-Institut, Federal Research Institute of Nutrition and Food, Department of Safety and Quality of Fruit and Vegetables, Haid-und-Neu-Straße 9, 76131, Karlsruhe, Germany; c School of Animal and Range Science, Haramaya University, P.O. Box 138, Dire Dawa, Ethiopia; d Ethiopian Agricultural Research Council Secretariat, P.O. Box 8115, Addis Ababa, Ethiopia

**Keywords:** Food microbiology, Bacteriology, Agricultural plant products, Food science, Bacteria involved in fermentation, Food fermentation, *Lactobacillus*, *Acetobacter*, High-throughput sequencing, Volatile compounds

## Abstract

Kocho is a traditional product in Ethiopia, prepared by fermenting parts of ‘false banana’ plants (*Ensete ventricosum*). Fermentation practices of kocho vary depending on the region of Ethiopia. In this study, 14 kocho samples originating from four different areas were investigated. They varied both in the fermentation technique and the duration of fermentation. Samples were analysed to determine the microbial community using culture-independent 16S amplicon high-throughput sequencing. In addition, bacterial strains were isolated and identified. Furthermore, the volatile profiles were characterized by HS-SPME treatment coupled with GC/MS. The results indicated that *Lactobacillus* and *Acetobacter* were the most dominant genera during kocho fermentation with *Lactobacillus plantarum* and *Lactobacillus brevis* being the prevalent species of *Lactobacillus.* The analysis of the volatile profiles demonstrated that acetic acid and butanoic acid prevailed in all samples. Our results showed that kocho samples prepared in different areas and using different processing methods varied both in the composition of the microbiota and in their volatile profiles.

## 1 Introduction

Kocho is a traditional fermented food product in Ethiopia and is produced by fermentation of parts of the ‘false banana’ (*Ensete ventricosum*). Kocho is prepared from the pseudostem and corm which is scraped and fermented (solid state fermentation) (National Research Council, 2006). The fermentation method and duration differs from area to area, sometimes even from household to household. According to Hunduma and Ashenafi (2011), fermentation is carried out in a pit after supplementation of a traditional starter for about 2–5 months in regions at high altitude; while in regions at low altitude traditional surface fermentation is followed by pit fermentation as a two-step process continued for about 2–4 months. Traditional surface fermentation for 2–4 weeks by supplementation of a traditional starter is common in the Gedio zone (Tsegaye and Gizaw, 2015).

*Ensete ventricosum* is an efficient crop compared to other crops in Ethiopia concerning edible dry weight and energy per area and time (Tsegaye and Struik, 2001). Moreover, cultivation of *E. ventricosum* has further advantages, e.g. cultivation under a broad range of environmental and soil conditions, low cultivation costs and availability throughout the year (Bosha et al., 2016). However, kocho is hallmarked by a distinctive odor. The causes are not fully characterized yet, but Urga et al. (1997) suggested that the activity of *Clostridium* species induces a butyrous smell.

Lactic acid bacteria (LAB), *Enterobacteriaceae*, spore forming bacteria and yeast are reported as responsible microbes for acid production during kocho fermentation (Karssa et al., 2014; Bosha et al., 2016). Nevertheless, these studies are mainly based on microbiological counts determined on agar media and phenotypic identification of randomly selected isolates. The objectives of our study were to characterize the microbiota, to determine the profiles of volatile organic components and to investigate the correlation of the diversity of the microbiota and volatile profiles.

## 2 Materials and methods

### 2.1 Sample collection

A number of 14 kocho samples were collected from four different areas in Ethiopia (Wolkite, n = 7; Dilla, n = 3; Ginchi, n = 2 and Woliso, n = 2). The samples differed in the fermentation techniques as well as in the fermentation periods (Table 1 and dataset (Stoll et al., 2019)). The fermentation periods of samples ranged from 7 days to 10 months. In Wolkite and Ginchi, the surface fermentation technique was used for the first nine days and three weeks, respectively, followed by pit fermentation for several months. In Woliso, pit fermentation was carried out for about 2–3 months with the addition of traditional starter cultures after the first 15 days of fermentation. In Dilla, a starter culture was added immediately and surface fermentation was carried out for about two weeks. In the case of Wolkite samples, a small succession study could be performed as five samples were collected from the same household (household A) at different time points. In Dilla, two samples were collected from the same household (household C) at different time points. Samples originating from Woliso were purchased from the same local market at different time points. All samples were collected in sterile plastic bags, immediately cooled and transferred to Addis Ababa University.

**Table 1:** Overview of kocho samples, sampling areas and fermentation types.

Sample number	Sampling area	Sampling place	Fermentation period	Fermentation type
W1	Wolkite (South-western Ethiopia)	Household A	9 days + 10 months	Surface fermentation followed by pit fermentation
W3		Household A	9 days + 9 months	
W6		Household B	9 days + 8 months	
W7		Household B	9 days + 7 months	
W8		Household A	9 days + 21days	
W9		Household A	9 days + 11 days	
W10		Household A	9 days + 1days	
D1	Dilla (South Ethiopia)	Household C	7 days	Surface fermentation with immediate addition of starter cultures (backslopping)
D2		Household C	14 days	
D3		Local market	approx. 2 weeks^a^	

G2	Ginchi (Western Ethiopia)	Household D	3 weeks + 3 months	Surface fermentation, followed by pit fermentation
G3		Household D	3 weeks + 1 week	
Wol-1	Woliso (South-western Ethiopia)	Local market	approx. 3 months^b^	Pit fermentation with the addition of starter cultures (backslopping)
Wol-2				

a In general, the fermentation period in Dilla lasts 2 weeks. As this sample was collected from a local market the exact fermentation period is unknown.

b In general, the fermentation period in Woliso lasts between 2-3 months. As these samples were collected from a local market the exact fermentation period is unknown.

### 2.2 Enumeration and isolation of microbes

A number of 10 kocho samples from Wolkite (n = 6), Dilla (n = 3), and Woliso (n = 1) were used for enumeration and isolation of microbes (see dataset (Stoll et al., 2019)). Serial dilutions (up to 10^−7^) of 10 g of kocho samples were used for enumerations of LAB; aerobic, mesophilic bacteria; enterobacteria; as well as yeast using the following agar media: de Man, Rogosa and Sharpe (MRS), plate count agar, violet red bile dextrose (VRBD) supplemented with 1% glucose, and potato dextrose (PDA), respectively (Hi Media, Mumbai, India). Samples were homogenized with 90 ml sterile distilled water using orbital shaker at 250 rpm for 30 min. A volume of 0.1 ml of the diluted sample was spread onto the agar plates in duplicates. All plates were incubated at 30 °C for 48 h, except for yeast which were incubated at 28 °C for 72 h. For characterization of LAB, colonies were selected randomly from plates of dilutions 10^−6^ and 10^−7^ and repeatedly streaked out until purity. Cultures were stored in MRS broth containing 15% of glycerol at -80 °C.

### 2.3 Phenotypic characterization

Cell morphology was observed by phase contrast microscopy at 1000x magnification. Strains were Gram-stained; catalase and oxidase activity and production of CO_2_ from glucose was determined using standard microbiological methods. The configuration of d (-) and l (+) isomers of lactic acid produced from glucose was determined enzymatically using a commercial test kit (R-biopharm, Darmstadt, Germany).

### 2.4 Genotypic characterization

The total genomic DNA of all strains was isolated using the DNeasy Blood and Tissue kit (Qiagen, Hilden, Germany) according to the manufacturer's instructions. The almost complete 16S rRNA gene of all strains was amplified by PCR, bi-directionally sequenced and analyzed as described by Danylec et al. (2018).

For culture-independent analysis, DNA of 200 mg of kocho samples was isolated using NucleoSpin DNA Stool Kit (Macherey-Nagel, Germany) as indicated by the manufacturer. The DNA concentration was measured using a Qubit 2.0 fluorometer (Thermo Fisher Scientific) and stored at -20 °C until analysis. The 16S amplicon high-throughput sequencing library preparation was based on the 16S Library Prep Guide released by Illumina Inc (2013) targeting the V3/V4 region with primer sequences published by Klindworth et al. (2013). Deviating from these instructions, the amplicon PCR was performed using ALLin HotStart Taq Mastermix, 2x (HighQu, Kraichtal, Germany) in 25 μl total reaction volume. Each primer volume was 1 μl (10 pmol/μl). Template DNA was 50 ng, if the starting concentration was too low the maximal possible amount was used. Amplifications were conducted in a peqSTAR 96 Universal Thermocycler (VWR International GmbH, Darmstadt, Germany) with the following program: 1 min at 95 °C, 20 cycles of 15 sec at 95 °C, 15 sec at 55 °C and 15 sec at 72 °C. To confirm successful amplification, 5 μl of PCR products were subjected to gel electrophoresis. The remaining 20 μl were cleaned up using Mag-Bind® RxnPure Plus Magnetic Beads (Omega Bio-tek Inc., Norcross, USA). Index PCR was conducted in a total volume of 50 μl using 0.5 μl Phusion Hot Start Flex DNA Polymerase, 10 μl buffer HF (New England BioLabs Inc., Ipswich, USA), 8 μl 1.25 mM dNTPs (VWR International GmbH), 5 μl of purified PCR product and 5 μl of each Index Primer. The PCR program was chosen as follows: 3 min at 98 °C, 8 cycles of 30 sec at 98 °C, 30 sec at 55 °C, 15 sec at 72 °C and 5 min at 72 °C. After a second clean up, DNA was quantified using the Qubit® dsDNA HighSensitivity Assay Kit (Thermo Fisher Scientific Inc.) on a Qubit 2.0 fluorometer. A 300 nt paired-end sequencing run was conducted on a MiSeq benchtop sequencing system (Illumina) according to the manufacturer's instructions. Single samples were normalized to a final concentration of 4 nM and afterwards pooled for parallel high-throughput sequencing. For quality issues and as internal control, 20% PhiX was supplemented to the final sequencing library.

### 2.5 Volatile profiling using GC–HS–SPME-MS

**Fig. 1:**
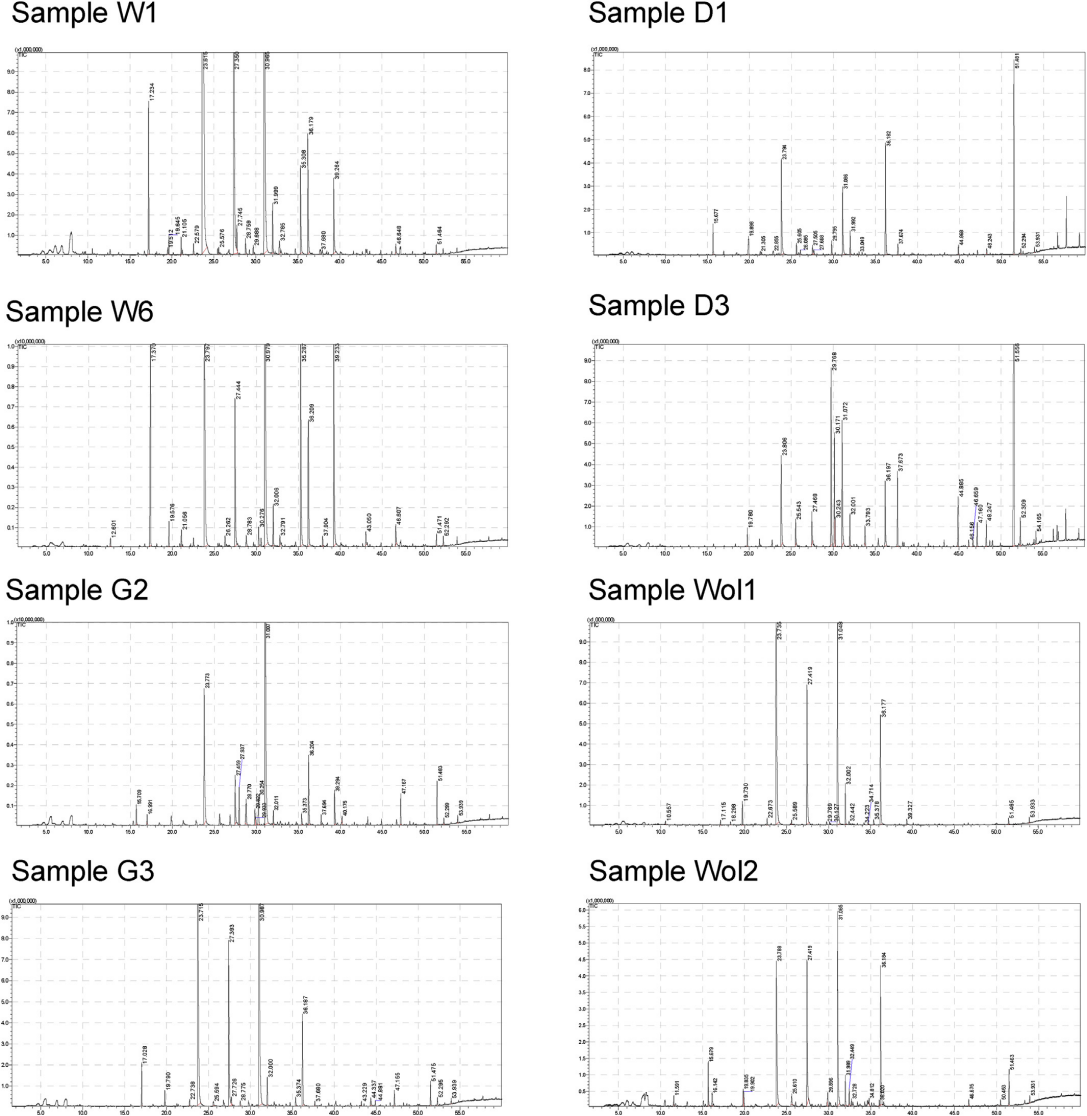
Exemplary chromatograms of the volatile profiling of different kocho samples.

One gram of the sample was placed in a 10 ml screw-capped vial. 0.1 g sodium chloride (Sigma-Aldrich, Taufkirchen, Germany), 50 μl of an aqueous solution of 2-ethyl butanoic acid (Sigma-Aldrich, 1 μg/μl) as internal standard, 500 μl of methanol (Carl Roth, Karlsruhe, Germany) and 500 μl of ultra-pure water were added and mixed by vortexing. Volatile profiling was done on a GCMS QP2010 ultra gas chromatography-mass spectrometry system equipped with an AOC-5000 autosampler (Shimadzu, Kyoto, Japan). A 50/30 DVB/CAR/PDMS fibre (Supelco, 57329-U) was used for the extraction of volatile organic compounds. In the autosampler, samples were first incubated in the agitator at 60 °C for 60 sec at 250 rpm. Afterwards, the SPME fibre was exposed to the headspace and extraction was carried out for 30 min. Desorption in the GC injection port was performed for 5 min at 250 °C in splitless mode (splitless time 0.5 min) and finally, the fiber was regenerated for 15 min at 270 °C in the needle heater. GC separation was done on a 60 m × 0.25 mm i.d. ZB-wax plus column with a film thickness of 0.25 μm. The oven temperature was programmed from 50 °C (2 min hold) to 200 °C at 3 °C/min and finally increased to 230 °C at 10 °C/min (total run time: 65 min). Flow rate of the helium carrier gas was 1.9 ml/min (= 27.9 cm/sec). The MS was operated in EI (70 eV) scan mode and the ion source and interface temperatures were 200 °C and 230 °C, respectively. The chromatograms were recorded by monitoring the total ion currents in a mass range of 40–450 m/z. Sample preparation, HS-SPME extraction, and GC-MS analysis was performed in duplicate in separate batches. Exemplary chromatograms are presented in Fig. 1.

### 2.6 Data and statistical analyses

The sequence quality of 16S rRNA gene sequences of pure cultures was checked with Chromas (version 2.4.1, Technelysium). Sequences were edited using SeqBuilder, forward and reverse reads were aligned using MegAlign (version 10.1.1, DNASTAR). Sequences were subjected to BLAST search.

The 16S amplicon high-throughput sequencing raw reads were analyzed as follows: The obtained reads were trimmed using a custom Perl script. Reads were scanned for the forward and reverse primer sequences, which then were trimmed. Reads deviating at least in one base from the used primer sequences were erased from the data set. Afterwards, the remaining reads were processed using mothur 1.39.5 (Schloss et al., 2009) according to the MiSeq SOP (Kozich et al., 2013). Basically, bi-directional reads were merged into contigs, only sequences between 300 nt and 450 nt were used. Additionally, all sequences containing any ambiguous bases or homopolymer regions over 12 nt were excluded from further analysis. The sequences were collapsed in unique copies and aligned against the SILVA v128 database (Quast et al., 2013; Yilmaz et al., 2014). Performing preclustering, up to four mismatches were allowed. Chimera sequences were identified using VSEARCH algorithm (Rognes et al., 2016) and subsequently erased. Remaining sequences were classified taxonomically (Wang et al., 2007) and unknown sequences were erased from the dataset (Stoll et al., 2019) as well as sequences belonging to archaea, eukaryota, chloroplasts, and mitochondria. For OTU based analysis, sequences were clustered in OTUs at a 0.03 similarity cut-off.

For statistical analysis of the GC-MS data, principal component analysis (PCA) was carried out using JMP 13 software (SAS). In this study, a duplicate analysis of each sample on two measurement days was performed, the resulting peak areas were normalized to the internal standard as well the weighed amount of the samples ('biomass'). A number of 27 out of the 33 detected volatile compounds exhibited less than 30% missing values (i.e., non-detects) and thus were considered for PCA after replacing the missing values by the minimal value of the respective column.

## 3 Results and discussion

For the analysis of kocho in this study, samples were collected from four different areas in Ethiopia differing in the fermentation technique and length. As this study was intended as a pilot study including microbiological, molecular biological and analytical methods the number of samples is limited.

The methods that were used included culture-dependent as well as culture-independent approaches to characterize the microbiota and volatile profiling. Using a combination of these methods enabled an insight into the fermentation process of kocho.

According to results of culture-dependent methods LAB and aerobic, mesophilic bacteria constituted the dominant microorganisms (see dataset (Stoll et al., 2019)). Within the first 30 days of fermentation, the counts of LAB were in the range of 10^7^–10^8^ CFU/g (samples W8, W9 and W10) and decreased to 10^2^–10^3^ CFU/g when fermentation was prolonged to 8 months and longer (samples W1, W3 and W6). Comparable to the counts of LAB, the counts of aerobic, mesophilic bacteria were higher within the first 30 days with counts of ca. 10^4^ CFU/g (samples W8, W9 and W10) in contrast to the longer fermented kocho samples (W1, W3 and W6) with counts of ca. 10^2^–10^3^ CFU/g. In the case of yeast and enterobacteria, comparatively higher counts were detected at the first fermentation week and decreased to below the detection limit during fermentation. Our results are in good agreement with the literature as other authors also stated that LAB were the most abundant microbes at the first stages of kocho fermentation and remained dominant until the end of fermentation with a slight decrease at the later stages (Muyanja et al., 2003; Andeta et al., 2018).

A number of 93 bacterial strains were isolated on MRS agar and characterized using phenotypic and genotypic methods (Table 2). All strains were Gram-positive, rod-shaped, oxidase- and catalase-negative, except for four strains which were Gram-negative, catalase-positive and later identified as acetic acid bacteria (AAB) *Acetobacter pasteurianus* and *A. persici/farinalis/malorum*.

**Table 2:** Number, origin and identification of bacterial strains isolated on MRS agar using phenotypic methods and 16S rRNA gene sequencing.

Identification	Total (n = 93)	W1	W3	W6	W8	W9	W10	D1	D2	D3	Wol-1
*L. plantarum*-group	45	2	2	5	6	5	2	2	6	5	10
*L. brevis*	31	2	2	-	1	3	6	6	5	2	4
*L. fermentum*	3	-	-	-	3	-	-	-	-	-	-
*L. paracasei*	9	2	1	-	-	-	2	-	-	1	3
*L. paracollinoides/collinoides*	1	-	1	-	-	-	-	-	-	-	-
*A. pasteurianus*	3	-	3	-	-	-	-	-	-	-	-
*A. persici/farinalis/malorum*	1	-	-	-	-	-	-	-	1	-	-

Thus, in total a number of 89 LAB strains were isolated from kocho samples. Among those, 35 strains were obligately heterofermentative producing dl-lactate, except one strain which produced d-lactate, and 54 were facultatively heterofermentative producing l- and dl-lactate. Within the group of obligately heterofermentative strains the majority was identified with 16S rRNA gene sequencing as *L. brevis* (n = 31), few strains as *L. fermentum* (n = 3) and one strain as *L. paracollinoides/collinoides*. The group of facultatively heterofermentative strains consisted of strains belonging to the *L. plantarum*-group (n = 45) and *L. paracasei* (n = 9). The *L. plantarum*-group consists of the species *L. plantarum, L. paraplantarum* and *L. pentosus* which cannot be separated by phenotypic methods and 16S rRNA gene sequencing reliably (Hammes and Hertel, 2006).

**Fig. 2:**
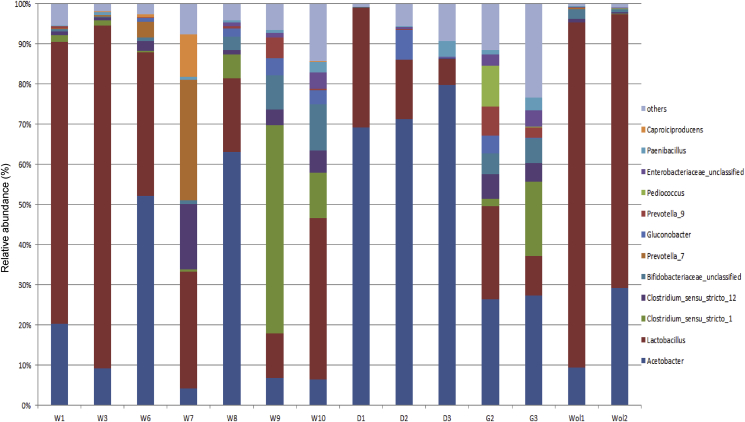
Analysis of the microbial community of the fermenting kocho samples at genus level. The analysis was performed using 16S rRNA gene NGS amplicon high-throughput sequencing.

Using culture-independent 16S amplicon sequencing, AAB and LAB could be identified as the most dominant bacterial groups within all kocho samples (Fig. 2). OTUs assigned to *Acetobacter* could be detected in every sample. The microbiota of samples from Dilla was mostly (ca. 70%–80%) dominated by *Acetobacter.* In addition, reads could also be assigned to *Gluconobacter* which was also present in each sample with the exception of Woliso samples. The higher abundance of AAB at the early stages of the fermentation of Dilla samples could be due to the addition of the starter cultures immediately at the beginning of the fermentation. The starter culture in this area is mainly prepared from the corm in combination with a mixture of rotten pseudostem and banana fruit, or previously fermented kocho. *Acetobacter* spp. were frequently isolated from rotten banana fruit and parts of plants (Samuel et al., 2016). AAB are described to be involved in different spontaneously food fermentations, e.g. cocoa (De Roos and De Vuyst, 2018). The main reason for the occurrence of AAB during cocoa fermentation is due to the higher presence of air in the fermenting mass when the pulp is drained away (Lefeber et al., 2010). During kocho fermentation, air may also be entered into fermenting mass during continuous opening of the cover to mixing, turning and chopping (Bosha et al., 2016) which creates suitable conditions for those microbes. To the best of our knowledge, this is the first time that AAB strains were isolated from kocho.

LAB was the second most abundant bacterial group. The microbiota of most Wolkite samples was dominated by *Lactobacillus*, which occurred in all samples in varying abundances. In the literature, the participation of LAB in kocho fermentation is well described (Bosha et al., 2016; Andeta et al., 2018). Additionally, *Clostridium*, enterobacteria, *Bifidobacterium,* and *Prevotella* could also be detected in kocho samples, comparable to the study of Andeta et al. (2018).

Moreover, *Caproiciproducens* was detected in all Wolkite samples with highest relative abundances in the samples W6 and W7. The microbial diversity of samples stemming from the same area seemed to be closely related to each other. The microbial succession study of Wolkite samples obtained from household A indicates that *Lactobacillus*, which was present from the beginning, became more dominant during the fermentation progress. These results indicated that the microbial diversity of kocho depends on the length of fermentation time, technique and region.

The volatile compounds, which are mostly produced by microbial activity in fermented food, contribute to a large extent to the flavor and odor of these foods (Salmerón et al., 2013; Nwokeleme and Ugwuanyi, 2015). In total, 27 volatile organic compounds were consistently detected and relatively quantified in the kocho samples (see dataset (Stoll et al., 2019) and Table 3). Methyl esters were the dominant ester type. Notably, one sample from Dilla local market (D3) exhibited a high amount of different forms of methyl esters like C10:0, C12:0 and C16:0 (see dataset (Stoll et al., 2019)). During fermentation, methyl esters are described to be produced enzymatically by methanolysis of acyl-CoA that is formed during fatty acid synthesis or degradation (Nwokeleme and Ugwuanyi, 2015). Methyl esters have been reported as responsible volatile aroma compounds in fermented food produce (Perestrelo et al., 2006; Ohiri and Bassey, 2017).

**Table 3:** Identification of volatile analytes (n = 27) of kocho samples.

Peak Name	Compound name	RT (min)	MSI level^a^	Similarity^b^
A01	Hexanoic acid methyl ester	12.804	2	92%
A03	Unknown01	15.707	4	
A04	Heptanoic acid methyl ester	16.88	2	92%
A05	Acetoin (3-Hydroxy-2-butanone)	17.074	2	96%
A06	Octanoic acid methyl ester	21.214	2	94%
A07	Acetic acid	23.688	2	95%
A08	Nonanoic acid methyl ester	25.547	2	92%
A09	Propanoic acid	27.399	2	96%
A10	2.3-Butanediol	27.718	2	95%
A12	Isobutyric acid	28.782	2	95%
A13	Decanoic acid methyl ester	29.751	2	94%
A16	Butanoic acid	31.024	2	93%
A18	Isovaleric acid	32.806	2	95%
A19	Unknown05	33.039	4	
A20	Pentanoic acid	35.328	2	96%
A22	Dodecanoic acid methyl ester	37.666	2	94%
A23	Hexanoic acid	39.292	2	92%
A25	Heptanoic acid	43.082	2	93%
A26	Fatty acid methyl ester (probably branched)	43.224	3	
A27	Tetradecanoic acid methyl ester	44.871	2	93%
A28	Octanoic acid	46.646	2	95%
A29	Methyltetradecanoic acid or isomer	46.65	3	85%
A30	12-Methyltetradecanoic acid	47.156	2	94%
A31	Pentadecanoic acid methyl ester	48.237	2	95%
A32	Hexadecanoic acid methyl ester	51.466	2	95%
A33	Unknown05	52.288	4	
A34	Unknown06	53.154	2	

a MSI identification levels: 1, compound unequivocally identified by spiking a sample with an analytical standard; 2, compound identified by matching against the NIST2014 spectral library; 3, compound or compound class tentatively identified based on spectral similarity or specific masses; 4, unknown compound.

b National Institute of Standards and Technology (NIST) Database 2014 was used.

**Fig. 3:**
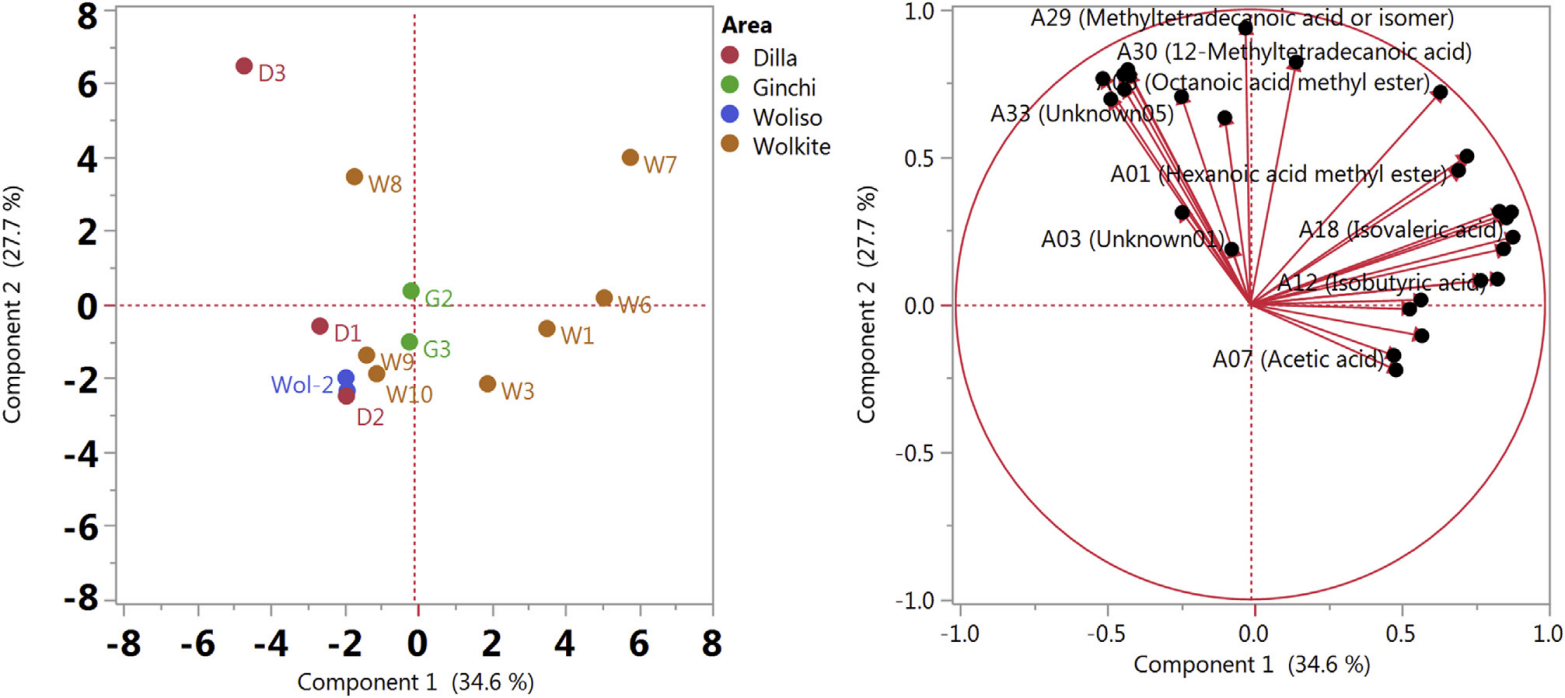
Principle Component Analysis: Scores plot showing similarities and differences of the volatile profiles of the 14 kocho samples (left); loadings plot highlighting the analytes contributing most to the separation (right). Analytes with more than 30% missing values were excluded.

A variety of carboxylic acids was detected in relatively high abundance in all kocho samples, especially acetic acid, propanoic acid, butanoic acid, pentanoic acid and hexanoic acid. As shown by PCA (Fig. 3), the volatile profiles of the Wolkite samples were more variable. The short-term fermented Wolkite samples had similar profiles to the Dilla and Woliso samples which were also fermented for a short period of time. The longer fermented Wolkite samples (7–10 months) were characterized by higher levels of short-chain fatty acids. Gashe (1987) stated that during fermentation of kocho a strong butyrous smell is common which could be confirmed by our study and was probably caused by the high abundance of short chain fatty acids. Butanoic acid was the dominant carboxylic acid in samples obtained from Wolkite and Ginchi. One explanation could be the occurrence of a higher abundance of *Clostridium* strains in those samples (Fig. 2). *Clostridium* is known to convert lactic acid to butanoic acid at low pH values (Wiegel et al., 2006). Butanoic acid has a persistent, penetrating, rancid, butter-like odor (Smit et al., 2005). Acetic acid was the second abundant compound in all kocho samples, probably due to the relatively high quantity of *Acetobacter*. AAB as *Acetobacter* oxidize ethanol and lactic acid to acetic acid and acetoin (De Roos and De Vuyst, 2018). Acetic and lactic acid are the primary metabolic products of LAB. However, lactic acid could not be detected in any kocho sample, probably due to the comparatively low volatility of this compound (Kam et al., 2011). Moreover, lactate can be catabolized by some lactobacilli such as *L. brevis, L. buchneri* and *L. plantarum* under anaerobic conditions (Elferink et al., 2001; Kam et al., 2011). Long fermentation time may have offered enough time for degradation of lactate, and for production of acetic acid, instead of lactic acid (Kam et al., 2011).

Further, relatively high abundances of hexanoic acid were detected in the longer fermented Wolkite samples and especially the samples W6 and W7. This could be due to the presence of *Caproiciproducens* species which we detected in the culture-independent approach of those samples (Fig. 2). *Caproiciproducens* species are strictly anaerobic bacteria and are described to produce hexanoic acid (Kim et al., 2017). Similar to other short chain fatty acids, hexanoic acid has a sickening, sweaty, rancid sour, sharp, pungent and unpleasant odor (Smit et al., 2005). In addition, 2,3-butanediol and 3-hydroxy-2-butanone occurred in almost all kocho samples. 2,3-butanediol has a cream-like odor and is produced by a range of sugar-fermenting microbes; 3-hydroxy-2-butanone has a bland, woody, yoghurt odor and a fatty creamy butter taste (Köpke et al., 2011). The presence of enterobacteria in all kocho samples could be a reason for the production of this volatile compound as enterobacteria are known to ferment d-glucose to a variety of organic acids, acetone and 2,3-butanediol, depending on the environmental conditions (Geckil et al., 2004).

## 4 Conclusion

Our results indicated that kocho samples prepared in different areas and by different processing methods varied both in the composition of the microbiota and in the quantity of volatile profiles. Besides AAB and LAB that presented the dominant microbiota in fermenting kocho, *Clostridium, Bifidobacterium,* and *Prevotella* were present. *Lactobacillus plantarum* and *L. brevis* were found to be the most abundant LAB species and could be detected in all samples from the beginning to the later stages of kocho fermentation. The volatile compounds which lead to the characteristic flavor and odor of kocho could be identified as short chain fatty acids which were presumably produced by microbial activity.

## Declarations

### Author contribution statement

Helen Weldemichael: Conceived and designed the experiments; Performed the experiments.

Dominic Stoll, Christoph Weinert: Performed the experiments; Analyzed and interpreted the data; Wrote the paper.

Tesfemariam Berhe: Contributed reagents, materials, analysis tools or data.

Shimelis Admassu, Melaku Alemu: Conceived and designed the experiments.

Melanie Huch: Conceived and designed the experiments; Analyzed and interpreted the data; Wrote the paper.

### Funding statement

This work was supported by Addis Ababa University, Institute of Technology, Ethiopia and the Swedish International Development Cooperation Agency (SIDA).

### Competing interest statement

The authors declare no conflict of interest.

### Additional information

Data associated with this study has been deposited at OpenAgrar under the DOI: 10.25826/Data20180720-110520.

## References

[bib1] Andeta A.F., Vandeweyer D., Woldesenbet F., Eshetu F., Hailemicael A., Woldeyes F. (2018). Fermentation of enset (*Ensete ventricosum*) in the Gamo highlands of Ethiopia: physicochemical and microbial community dynamics. Food Microbiol..

[bib2] Bosha A., Dalbato A.L., Tana T., Mohammed W., Tesfaye B., Karlsson L.M. (2016). Nutritional and chemical properties of fermented food of wild and cultivated genotypes of enset (*Ensete ventricosum*). Food Res. Int..

[bib3] Danylec N., Goebl A., Stoll D.A., Hetzer B., Kullling S.E., Huch M. (2018). *Rubneribacter badeniensis* gen. nov., sp. nov. and *Enteroscipio rubneri* gen. nov., sp. nov., new members of the *Eggerthellaceae* isolated from human faeces. Int. J. Syst. Evol. Microbiol..

[bib4] De Roos J., De Vuyst L. (2018). Acetic acid bacteria in fermented foods and beverages. Curr. Opin. Biotechnol..

[bib5] Elferink S.J.O., Krooneman J., Gottschal J.A., Spoelstra S.F., Faber F., Driehuis F. (2001). Anaerobic conversion of lactic acid to acetic acid and 1,2-propanediol by *Lactobacillus buchneri*. Appl. Environ. Microbiol..

[bib6] Gashe B.A. (1987). Kocho fermentation. J. Appl. Bacteriol..

[bib7] Geckil H., Barak Z., Chipman D.M., Erenler S.O., Webster D.A., Stark B.C. (2004). Enhanced production of acetoin and butanediol in recombinant *Enterobacter aerogenes* carrying *Vitreoscilla* hemoglobin gene. Bioproc. Biosyst. Eng..

[bib8] Hammes W.P., Hertel C., Dworkin M., Falkow S., Rosenberg E., Schleifer K.H., Stackebrandt E. (2006). The genera *Lactobacillus* and *Carnobacterium*. The Prokaryotes.

[bib9] Hunduma T., Ashenafi M. (2011). Effect of altitude on microbial succession during traditional enset (*Ensete ventricosum*) fermentation. Int. J. Food Saf. Nutr. Public Health.

[bib10] Illumina Inc (2013). 16S Metagenomic Sequencing Library Preparation - Preparing 16S Ribosomal RNA Gene Amplicons for the Illumina MiSeq System.

[bib11] Kam W.Y., Aida W.W., Sahilah A.M., Maskat M.Y. (2011). Volatile compounds and lactic acid bacteria in spontaneous fermented sourdough. Sains Malays..

[bib12] Karssa T.H., Ali K.A., Gobena E.N. (2014). The microbiology of Kocho: an ethiopian traditionally fermented food from enset (*Ensete ventricosum*). Int. J. Life Sci..

[bib13] Kim B.C., Jeon B.S., Kim S., Kim H., Um Y., Sang B.I. (2017). *Caproiciproducens galactitolivorans* gen. nov., sp. nov., a bacterium capable of producing caproic acid from galactitol, isolated from a wastewater treatment plant. Int. J. Syst. Evol. Microbiol..

[bib14] Klindworth A., Pruesse E., Schweer T., Peplies J., Quast C., Horn M., Glöckner F.O. (2013). Evaluation of general 16S ribosomal RNA gene PCR primers for classical and next-generation sequencing-based diversity studies. Nucleic Acids Res..

[bib15] Köpke M., Mihalcea C., Liew F., Tizard J.H., Ali M.S., Conolly J.J. (2011). 2, 3-Butanediol production by acetogenic bacteria, an alternative route to chemical synthesis, using industrial waste gas. Appl. Environ. Microbiol..

[bib16] Kozich J.J., Westcott S.L., Baxter N.T., Highlander S.K., Schloss P.D. (2013). Development of a dual-index sequencing strategy and curation pipeline for analyzing amplicon sequence data on the MiSeq Illumina sequencing platform. Appl. Environ. Microbiol..

[bib17] Lefeber T., Janssens M., Camu N., De Vuyst L. (2010). Kinetic analysis of strains of lactic acid bacteria and acetic acid bacteria in cocoa pulp simulation media toward development of a starter culture for cocoa bean fermentation. Appl. Environ. Microbiol..

[bib18] Muyanja C.M.B.K., Narvhus J.A., Treimo J., Langsrud T. (2003). Isolation, characterisation, and identification of lactic acid bacteria from bushera: a Ugandan traditional fermented beverage. Int. J. Food Microbiol..

[bib19] National Research Council (2006).

[bib20] Nwokeleme C.O., Ugwuanyi J.O. (2015). Evolution of volatile flavour compounds during fermentation of african oil bean (*Pentaclethra macrophylla* Benth) seeds for “ugba” production. Int. J. Food Sci..

[bib21] Ohiri R.C., Bassey E.E. (2017). Fermentation induced changes in volatile components of african oil bean (*Pentaclethra macrophylla* Benth) seeds. Food Sci. Nutr..

[bib22] Perestrelo R., Fernandes A., Albuquerque F.F., Marques J.C., Câmara J.D.S. (2006). Analytical characterization of the aroma of Tinta Negra Mole red wine: identification of the main odorants compounds. Anal. Chim. Acta.

[bib23] Quast C., Pruesse E., Yilmaz P., Gerken J., Schweer T., Yarza P. (2013). The SILVA ribosomal RNA gene database project: improved data processing and web-based tools. Nucleic Acids Res..

[bib24] Rognes T., Flouri T., Nichols B., Quince C., Mahé F. (2016). VSEARCH: a versatile open source tool for metagenomics. PeerJ.

[bib25] Salmerón I., Rozada R., Thomas K., Ortega-Rivas E., Pandiella S.S. (2013). Sensory characteristics and volatile composition of a cereal beverage fermented with *Bifidobacterium breve* NCIMB 702257. Food Sci. Technol. Int..

[bib26] Samuel O., Lina J., Ifeanyi O. (2016). Production of vinegar from oil-palm wine using *Acetobacter aceti* isolated from rotten banana fruits. Univ. J. Biomed. Eng..

[bib27] Schloss P.D., Westcott S.L., Ryabin T., Hall J.R., Hartmann M., Hollister E.B. (2009). Introducing mothur : open-source, platform-independent, community-supported software for sescribing and comparing microbial communities. Appl. Environ. Microbiol..

[bib28] Smit G., Smit B.A., Engels W.J. (2005). Flavour formation by lactic acid bacteria and biochemical flavour profiling of cheese products. FEMS (Fed. Eur. Microbiol. Soc.) Microbiol. Rev..

[bib29] Stoll D.A., Weinert C.H., Weldemichael H., Huch M. (2019). Characterization of the Microbiota and Volatile Components of Kocho, a Traditional Fermented Food of Ethiopia.

[bib30] Tsegaye Z., Gizaw B. (2015). Community indigenous knowledge on traditional fermented enset product preparation and utilization practice in Gedeo zone. J. Biodivers. Ecol. Sci..

[bib31] Tsegaye A., Struik P.C. (2001). Enset (*Ensete ventricosum* (Welw.) Cheesman) kocho yield under different crop establishment methods as compared to yields of other carbohydrate-rich food crops. NJAS - Wageningen J. Life Sci..

[bib32] Urga K., Fite A., Biratu E. (1997). Natural fermentation of enset (*Ensete ventricosum*) for the production of kocho. Ethiop. J. Health Dev..

[bib33] Wang Q., Garrity G.M., Tiedje J.M., Cole J.R. (2007). Naive bayesian classifier for rapid assignment of rRNA sequences into the new bacterial taxonomy. Appl. Environ. Microbiol..

[bib34] Wiegel J., Tanner R., Rainey F.A., Dworkin M., Falkow S., Rosenberg E., Schleifer K.H., Stackebrandt E. (2006). An introduction to the family *Clostridiaceae*. The Prokaryotes.

[bib35] Yilmaz P., Parfrey L.W., Yarza P., Gerken J., Pruesse E., Quast C. (2014). The SILVA and "All-species living tree project (LTP)" taxonomic frameworks. Nucleic Acids Res..

